# Optimization of late gadolinium enhancement cardiovascular magnetic resonance imaging of post-ablation atrial scar: a cross-over study

**DOI:** 10.1186/s12968-018-0449-8

**Published:** 2018-05-03

**Authors:** Henry Chubb, Shadman Aziz, Rashed Karim, Christian Sohns, Orod Razeghi, Steven E. Williams, John Whitaker, James Harrison, Amedeo Chiribiri, Tobias Schaeffter, Matthew Wright, Mark O’Neill, Reza Razavi

**Affiliations:** 1 School of Biomedical Engineering and Imaging Sciences, King’s College London, St Thomas’ Hospital, Westminster Bridge Road, London, SE1 7EH UK; 2 Department of Cardiology, St Thomas’ Hospital, London, UK

**Keywords:** Atrial fibrillation, Cardiovascular magnetic resonance imaging, Catheter ablation, Atrium, Optimization, Late gadolinium enhancement

## Abstract

**Background:**

Cardiovascular magnetic resonance (CMR) imaging may be used to visualize post-ablation atrial scar (PAAS), and three-dimensional late gadolinium enhancement (3D LGE) is the most widely employed technique for imaging of chronic scar. Detection of PAAS provides a unique non-invasive insight into the effects of the ablation and may help guide further ablation procedures. However, there is evidence that PAAS is often not detected by CMR, implying a significant sensitivity problem, and imaging parameters vary between leading centres. Therefore, there is a need to establish the optimal imaging parameters to detect PAAS.

**Methods:**

Forty subjects undergoing their first pulmonary vein isolation procedure for AF had detailed CMR assessment of atrial scar: one scan pre-ablation, and two scans post-ablation at 3 months (separated by 48 h). Each scan session included ECG- and respiratory-navigated 3D LGE acquisition at 10, 20 and 30 min post injection of a gadolinium-based contrast agent (GBCA). The first post-procedural scan was performed on a 1.5 T scanner with standard acquisition parameters, including double dose (0.2 mmol/kg) Gadovist and 4 mm slice thickness. Ten patients subsequently underwent identical scan as controls, and the other 30 underwent imaging with a reduced, single, dose GBCA (*n* = 10), half slice thickness (*n* = 10) or on a 3 T scanner (*n* = 10). Apparent signal-to-noise (aSNR), contrast-to-noise (aCNR) and imaging quality (Likert Scale, 3 independent observers) were assessed. PAAS location and area (%PAAS scar) were assessed following manual segmentation. Atrial shells with standardised %PAAS at each timepoint were then compared to ablation lesion locations to assess quality of scar delineation.

**Results:**

A total of 271 3D acquisitions (out of maximum 280, 96.7%) were acquired. Likert scale of imaging quality had high interobserver and intraobserver intraclass correlation coefficients (0.89 and 0.96 respectively), and showed lower overall imaging quality on 3 T and at half-slice thickness. aCNR, and quality of scar delineation increased significantly with time. aCNR was higher with reduced, single, dose of GBCA (*p* = 0.005).

**Conclusion:**

3D LGE CMR atrial scar imaging, as assessed qualitatively and quantitatively, improves with time from GBCA administration, with some indices continuing to improve from 20 to 30 min. Imaging should be performed at least 20 min post-GBCA injection, and a single dose of contrast should be considered.

**Trial registration:**

Trial registry- United Kingdom National Research Ethics Service 08/H0802/68 – 30th September 2008.

**Electronic supplementary material:**

The online version of this article (10.1186/s12968-018-0449-8) contains supplementary material, which is available to authorized users.

## Background

Over the last two decades there has been a substantial increase in the implementation of catheter ablation for the treatment of atrial fibrillation (AF) [1]. Concurrently, advances in cardiovascular magnetic resonance (CMR) imaging have enabled clinically useful visualisation of the left atrial (LA) wall and ablation lesions [2–11]. However, arrhythmia recurrence rates post-ablation remain high, and many of these recurrences are secondary to pulmonary vein (PV) reconnection or linear lesion discontinuities following ineffective lesion formation [12]. Effective and reliable non-invasive assessment of post-ablation atrial scar (PAAS) provides a unique insight into lesion formation and may help guide further ablation procedures.

Late gadolinium enhancmenet (LGE) CMR acquisition techniques have been shown to be the most sensitive to detect PAAS [13], and can identify gaps in ablation lesion sets [14]. In 2009, Peters et al. demonstrated that AF recurrence post-ablation correlates with the extent of PAAS [15]. This finding was corroborated by Badger et al. in 2010, who found that complete PV encirclement by CMR-defined PAAS had a very high positive predictive value in identifying electrical isolation [3]. However, the clinical implementation of these findings is controversial. In patients requiring repeat AF ablation procedure, some groups have shown good correlation between CMR-derived lesion gaps and sites of successful re-isolation [7, 11], whilst others have shown the opposite [9, 16].

Consistent between all studies is the finding that complete encirclement of PVs by CMR-detected PAAS is a rare occurrence. 90–100% of patients do not have complete encirclement of all PVs [2, 3, 7, 11, 16], in the context of a recurrence rate in these studies of 30–50%. In some cases, electrical reconnection will not result in AF recurrence [12, 17], but on invasive assessment many of the veins remain electrically isolated, despite detection of gaps in CMR scar [7]. The specificity of PAAS gaps for electrical reconnection is low and therefore imaging techniques need to be optimized in order to maximize detection of effective ablation injury.

The core imaging sequences have remained relatively unchanged from those first proposed and evaluated by the Boston and Utah groups [5, 6]. For this, a gadolinium-based contrast agent (GBCA) is injected and the enhancing structures can be detected after a delay using an inversion recovery (IR) prepared 3D acquisition. The T_1_-weighted electrocardiogram (ECG)- and respiratory-navigated 3D turbo gradient echo sequence is widely available on most imaging platforms and moderately robust [18]. Novel sequences will continue to be developed and employed, but mainstream use of PAAS imaging in the medium term is highly likely to rely upon these conventional imaging techniques. However, the acquisition parameters vary widely between leading groups (see Table 1). Timing post- GBCA administration, scanner field strength, slice thickness and even GBCA dose differ.

**Table 1:** Post-ablation atrial scar imaging techniques utilised in leading centres worldwide

	Centre	Subjects	Timing post-gadolinium	GBCA Dose	Scanner Strength	Slice thickness
Badger et al. (2010) [3]	Utah, USA	144	15 min (repeated if ‘suboptimal’)	0.1 mmol/kg (Multihance)	1.5 T	2.5 mm
Taclas et al. (2010) [11]	Boston, USA	19	15-20 min	0.2 mmol/kg (Magnevist)	1.5 T	4 mm
Hunter et al. (2013) [8]	Imperial/Barts, London, UK	50	20 min	0.4 mmol/kg (Magnevist)	1.5 T	4 mm
Bisbal et al. (2014) [7]	Barcelona, Spain	15	25-30 min	0.2 mmol/kg (Gadovist)	3 T	2.5 mm
Fukumoto et al. (2015) [4]	John Hopkins, USA	20	10-32 min	0.2 mmol/kg (Magnevist)	1.5 T	2 mm
Harrison et al. (2015) [9]	King’s College London, UK	20	20 min	0.2 mmol/kg (Gadovist)	1.5 T	4 mm
Akoum et al. (2015) [2]	DECAAF, Multicentre	177	15 min	0.1–0.2 mmol/kg (Multiple agents)	1.5 T (9 centres) 3 T (5 centres)	2.5 mm

In-plane resolution is 1.25 × 1.25 mm or 1.3 × 1.3 mm for all centres. GBCA: gadolinium based contrast agent. Note relaxivities vary significantly between GBCAs: Multihance r_1_ = 6.3 L mmol^− 1^ s^− 1^ (1.5 T) r_1_ = 5.5 L mmol^− 1^ s^− 1^ 1 (3 T), Magnevist r_1_ = 4.1 L mmol^− 1^ s^− 1^ (1.5 T) r_1_ = 3.7 L mmol^− 1^ s^− 1^ 1 (3 T), Gadovist r_1_ = 5.2 L mmol^− 1^ s^− 1^ (1.5 T) r_1_ = 5.0 L mmol^− 1^ s^− 1^ (3 T) [22]

The measurement of improvement in imaging should ideally be referenced to a gold standard or hard clinical endpoint. For assessment of PAAS, no gold standard is readily available. Comparison to invasive voltage mapping is prone to registration errors. Furthermore, voltage does not entirely reflect scar formation [19] and varies according to the electrode characteristics used to perform voltage mapping [20, 21]. Alternatively, the clinical end-point of arrhythmia recurrence does not necessarily reflect a lack of PAAS in any given location [9]. This study, therefore, has sought to measure the improvement in imaging parameters through the assessment of conventional subjective and objective imaging quality markers in a prospective cross-over study.

## Methods

### Study population

Between January 2014 and January 2016, all patients undergoing routine CMR imaging prior to their first PV ablation procedure were approached to join the study. Forty subjects provided written and informed consent and the study was approved by the National Research Ethics Service (South London Research Ethics Committee reference 08/H0802/68). Exclusion criteria were contraindication to CMR imaging or prior allergic reaction to GBCA. Baseline demographics and comorbidities were documented at the initial scan.

**Fig. 1:**
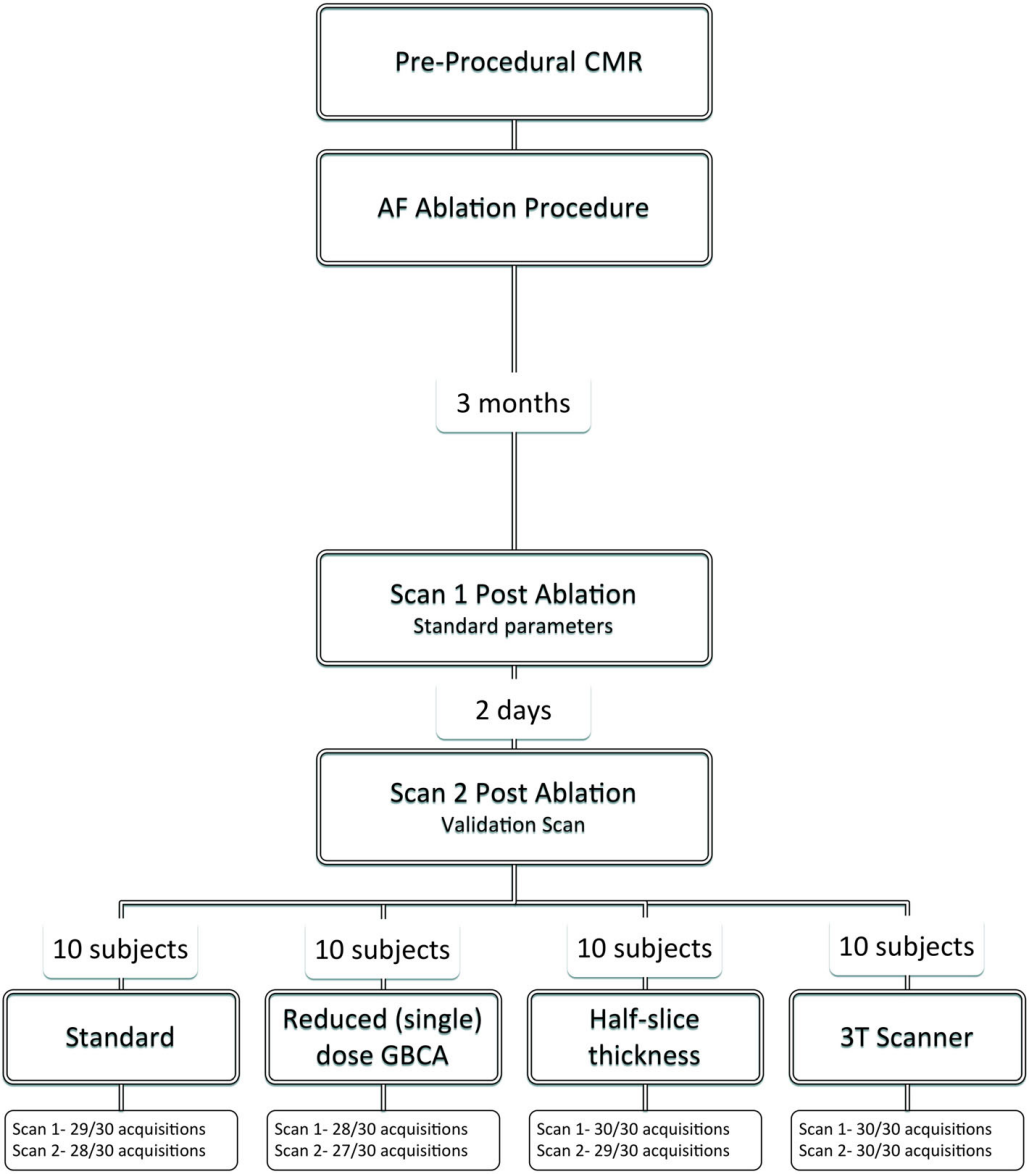
Flowchart demonstrating subject allocation and number of scan acquisitions achieved

All patients underwent CMR imaging on two occasions following clinically indicated catheter ablation for AF (Fig. 1) The first post-ablation CMR scan (Scan 1) was performed at approximately 3 months after the ablation procedure, regardless of rhythm or arrhythmia recurrence (median 94 days, (interquartile range (IQR) 89–101 days)), and was performed using standard acquisition parameters (see below). A second scan session (Scan 2) was performed approximately 2 days later (median 48.1 h, IQR 47.9–49.1 h). Subjects were allocated to scan 2 in 3 T scanner or the same 1.5 T scanner. 3 T scanner availability was limited, precluding randomization of allocation, but the allocation was performed without reference to patient outcome or demographics. The remaining patients were randomised in equal ratios to one of three different imaging parameter groups for scan 2: repeat scan with identical acquisition parameters, repeat with reduced, single, dose of GBCA, or repeat with half-slice thickness.

### CMR protocol

CMR imaging was performed on a 1.5 T CMR-scanner (Ingenia, Philips Healthcare, Best, the Netherlands), except for those allocated to 3 T scanner for scan 2 (see below). All patients underwent detailed assessment at pre-procedural CMR scan, including left ventricular (LV) and right ventricular (RV) volumes and function, LA volumes and function, and 3D LGE assessment of baseline LA fibrosis. Details of the methods used to quantify baseline LA fibrosis are available in the online supplement. Cine imaging was performed in an end-expiration breathhold using a standard multislice balanced steady state free precession (bSSFP) technique (effective TR 2.7 msec, TE 1.3 msec, 1.4 × 1.4 mm^2^ in-plane, slice thickness 10 mm, 50 phases). 3D inversion recovery spoiled gradient echo LGE acquisition was performed with coverage to include the entire LA in axial orientation. (TR 5.5 msec, TE 3.0 msec, flip angle 25°, low-high radial k-space ordering, respiratory and ECG-triggering (end atrial diastole, maximum 120 msec acquisition window, respiratory navigator leading with gating window 5 mm), 1.3 × 1.3 × 4 mm^3^ (typically 50 interpolated slices per acquisition, reconstructed to 0.94 × 0.94 × 2 mm^3^), SPIR fat suppression, pixel bandwidth 540 Hz, phase-encoding direction AP, parallel imaging: SENSE P-reduction (AP) factor 2, 32 channel phased array digital receiver coil). Average acquisition window onset was 296 ± 40 msec post R-wave, and end at 398 ± 39 msec, adjusted bespoke to each subject. GBCA dose was 0.2 ml/kg Gadovist (Bayer HealthCare Pharmaceuticals, Berlin, Germany).

Scan 1 (post-procedure) was performed using the same 3D LGE acquisition parameters as the baseline scan, and a total of three LA 3D LGE datasets were acquired, timed to start at 10 min, 20 min and 30 min after GBCA administration. The inversion time was determined from a Look-Locker acquisition performed immediately prior to each LGE acquisition to ensure nulling of the myocardium. In rare cases in which the acquisitions took longer than 10 min, the subsequent acquisition was started immediately.

Scan 2 (post-procedure) was performed with allocated modifications of the baseline scan, with acquisitions again performed at 10 min, 20 min and 30 min post GBCA administration.

1. **Reduced, single, gadolinium dose**. 0.1 mmol/kg of gadobutrol (Gadovist), otherwise unchanged
2. **Half-slice thickness**. The acquired voxel size was reduced to 1.3 × 1.3 × 2 mm (reconstructed 0.625 × 0.625x1mm). Field of view remained unchanged to cover the whole of the left atrium, and therefore approximately 90–100 slices were acquired, doubling the nominal acquisition duration.
3. **3 T scanner**. Scans were performed on 3 T scanner (Achieva, Philips Healthcare) with 32-channel coil. Parameters were matched to those for 1.5 T scanning as closely as possible (TR 4.0 msec, TE 2.0 msec, slice thickness 4 mm, pixel bandwidth 620 Hz, acquired voxel size 1.3 × 1.3 × 4 mm^3^).

Native T_1_-time constant assessment was performed prior to GBCA administration at each scan session in order to confirm myocardial washout. This was assessed using a bSSFP single breath-hold modified inversion recovery Look-Locker (MOLLI) sequence, in a single mid-LV short axis slice (TE: 1.64 ms, TR 3.3 ms, flip angle 50°, voxel size 1.8 × 1.8x8mm, phase encoding steps *n* = 166, 11 images from three inversions (3 + 3 + 5) with three heartbeat pauses prior to the second and third inversions and an adiabatic prepulse). Myocardial T_1_ relaxation was measured at the septal myocardium with T_1_ time constant extrapolated from the exponential model fitted using ViewForum workstation (Philips Healthcare) [22].

### Atrial fibrillation ablation protocol

Two experienced operators performed all catheter ablation procedures under general anaesthesia using Carto3 (Biosense Webster/Johnson&Johnson, New Brunswick, New Jersey, USA) electroanatomic mapping (EAM) system, with the exception of 8 procedures performed using EnSite Velocity (St Jude Medical, St Paul, Minnesota, USA). For patients with a diagnosis of paroxysmal AF and in sinus rhythm, a point-by-point wide area circumferential ablation (WACA) achieving PV isolation was performed using 8Fr irrigated SmartTouch catheter (Biosense Webster), or 8Fr irrigated TactiCath catheter (St Jude Medical). Target ablation parameters were > 5 g for at least 15 s per radiofrequency (RF) delivery location. Power was 30 W throughout except on the posterior wall, where it was limited to 25 W. Procedural endpoint was defined as PV isolation as confirmed on entry block (and exit block if capture achieved). For patients presenting with Persistant AF, a WACA was performed followed by additional ablation lesion sets (mitral line, roof line, inferior posterior line, complex fractionated electrogram ablation) as a step-wise ablation.

### Imaging assessment

#### Qualitative assessment

Qualitative assessment of all acquisitions was performed independently by three experienced observers (HC, JH, SA). Observers were presented with a single representative transverse slice at the level of the aortic root in random order, with 10 initial training sets, and 20 random acquisitions repeated in order to assess intra-observer reproducibility. Likert Scale assessment was performed, with acquisitions graded across four criteria: image sharpness, scar contrast, freedom from artefact and quality of myocardial nulling. All criteria were scored from 1 to 5, with a score of 5 indicating optimal imaging within each criterion, as well as an average of all four criteria.

#### Signal-to-noise and contrast-to-noise ratios

All acquisitions were analysed for signal-to-noise (SNR) and contrast-to-noise (CNR) ratios. In the presence of parallel imaging, noise is spatially heterogeneous throughout the imaging field, and should ideally be quantified through the assessment of multiple (> 10) identical acquisitions. However, this is not feasible on account of the substantial additional imaging time, and the shifting tissue signal intensities during the acquisition following GBCA administration.

Apparent SNR (aSNR) and apparent CNR (aCNR) were therefore calculated as the relationship between the mean signal intensity within a circular region of interest (ROI) within the blood (SI_Blood_), the mean of a ROI within scar (SI_Scar_), and the standard deviation of the background signal within the lungs (SD_L_) [23].

$$ {aSNR}_{Scar}=\frac{SI_{Scar}}{SD_L} $$

$$ {aSNR}_{Blood}=\frac{SI_{Blood}}{SD_L} $$

$$ aCNR={aSNR}_{Scar}-{aSNR}_{Blood} $$

ROIs were all selected within the same transverse slice, at the level of the origin of the left main coronary artery. For SI_Blood_, a 200mm^2^ circular ROI was placed in the LA blood pool, distant from potential artefact due to inflow enhanced by respiratory navigator signal; for SI_Scar_, a 5mm^2^ ROI within the most intense region of PAAS within slice, and for SD_L_ a 200mm^2^ ROI within the lung, distant from any apparent large blood vessels at the same distance from the surface coils as the blood pool ROI. It was noted that the ROI for scar was small, and the value was therefore validated against the highest intensity scar on the 3D shell (see below). For acquisitions performed at 10 min, the single ROI scar value was above a median of 98.8% (IQR 91.3–99.7%) of all atrial wall values for that acquisition. At 20 min, the figure was 99.4% (IQR 98.5–99.7%) and at 30 min 99.3% (IQR 98.8–99.8%).

### Scar quantification and quality of scar identification

Details of the method used to quantify LA scar are documented in the online supplement. In brief, the LA endocardial border was segmented from the 3D LGE dataset, using a high contrast gated magnetic resonance angiogram as a template. The scar was interrogated using a maximum intensity projection (MIP) technique (3 mm outside segmented shell, 1 mm within) and signal intensity values projected onto the 3D surface of the LA shell. For basic analysis, PAAS was thresholded at a single value of 3.3 standard deviations (SD) above the blood pool mean in accordance with histologically validated data [13], and expressed as a percentage of the total surface area of the LA (%PAAS). Further analysis was also performed in view of the confounding factors arising from the use of the single scar threshold: it is highly unlikely that a single threshold reflects the appropriate threshold for PAAS at all timings post GBCA administration. However, there is no published data to enable the rational selection of variable thresholds, and therefore the level of noise and quality of scar identification at equivalent thresholds at each timepoint was assessed.

Firstly, the %PAAS was identified for the 20 min post-GBCA acquisition at two published thresholds (3.3SD above the blood pool mean at 20 min post-GBCA [13], and image intensity ratio (IIR) 1.32 at 20 min post-GBCA [24]). Thresholds were then identified for the shells acquired at 10 min and 30 min post-GBCA in order to achieve the same scar burden (%PAAS). Finally, in order to assess the quality of scar identification, the point-by-point location of thresholded scar was compared to the location of ablation lesions. In a subset of 20 patients, the EAM shell (Carto3, Biosense Webster) was exported with objective lesion markers appended (VisiTag, Biosense Webster (thresholds: force 8 g, time 10s, 50% percentage time, range 3 mm, impedance drop and target temperature thresholds ‘off’ [25]). The CMR-derived shells were fused to the EAM shell using an iterative closest point technique, blinded to MR scar, and a Sorensen dice similarity coefficient (DSC) was used to analyse co-location of thresholded CMR LGE scar and ablation lesions [26].

$$ \kern3.5em DSC\left( Threshold(T)\Big)\right)=\kern0.5em \frac{2\left({Scar}_{CMR}\cap {Lesion}_{EAM}\right)}{Scar_{CMR}+{Lesion}_{EAM}} $$

where DSC (Threshold (T)) is the DSC for the comparison of the fused CMR shell with the ablation location EAM shell, at signal intensity threshold T. ScarCMR are the faces designated as scar on the CMR shell, at Threshold (T), and LesionEAM are the faces associated with an objective ablation marker (VisiTag).

A DSC was derived for each acquisition, using the threshold at 10 min and 30 min that gave the same %PAAS as the ‘standard’ threshold at 20 min. A high DSC represents good co-location of PAAS and ablation lesions, with lower DSC at fixed scar burden likely to represent inappropriate assignation of scar at locations distant to ablation.

### Statistical analysis

Data are expressed as mean (± standard deviation) for normally distributed data, and median (with interquartile range (IQR)) for non-normally distributed data. Statistical analysis was performed using SPSS Statistics (Version 22, International Business Machines, Armonk, New York, USA). Baseline parameters were compared using unpaired t-test for normally distributed continuous variables, Mann-Whitney U test for non-parametric variables and Χ^2^-test for categorical data. For Likert scores Intraclass correlation coefficient (ICC) was assessed using a two-way mixed effects model (average measures, alpha model). Assessment was for consistency for interobserver, and absolute agreement for intra-observer, and are quoted with 95% confidence interval (CI) [27], and two-way ANOVA was used to compare imaging parameters and timing of acquisition. Two-way ANOVA (repeated measures) was used to assess the effect of the scan parameters and timing of acquisition on aCNR and %PAAS area. The normality of the dependent variables was confirmed for all groups using Shapiro-Wilk’s test, except for the 10 min acquisitions for the ‘half-slice thickness’ group and the ‘3 T’ group (both *p* < 0.01). In order to exclude the strong time-related contribution of the acquisitions performed at 10 min, and to assess the change in imaging between acquisitions performed at 20 and 30 min, a further two-way ANOVA (repeated measures) was performed excluding the acquisitions at 10 min. The effect of timing of acquisition alone was further assessed for ‘standard’ acquisition parameters only: the Studentized residuals were not normally distributed (Shapiro-Wilk’s) and therefore Wilcoxon matched-pairs signed rank test was used to compare side-by-side time points, and Friedman test for overall effect of acquisition time. *P*-values are quoted following Bonferroni correction for multiple comparisons where applicable.

## Results

**Fig. 2:**
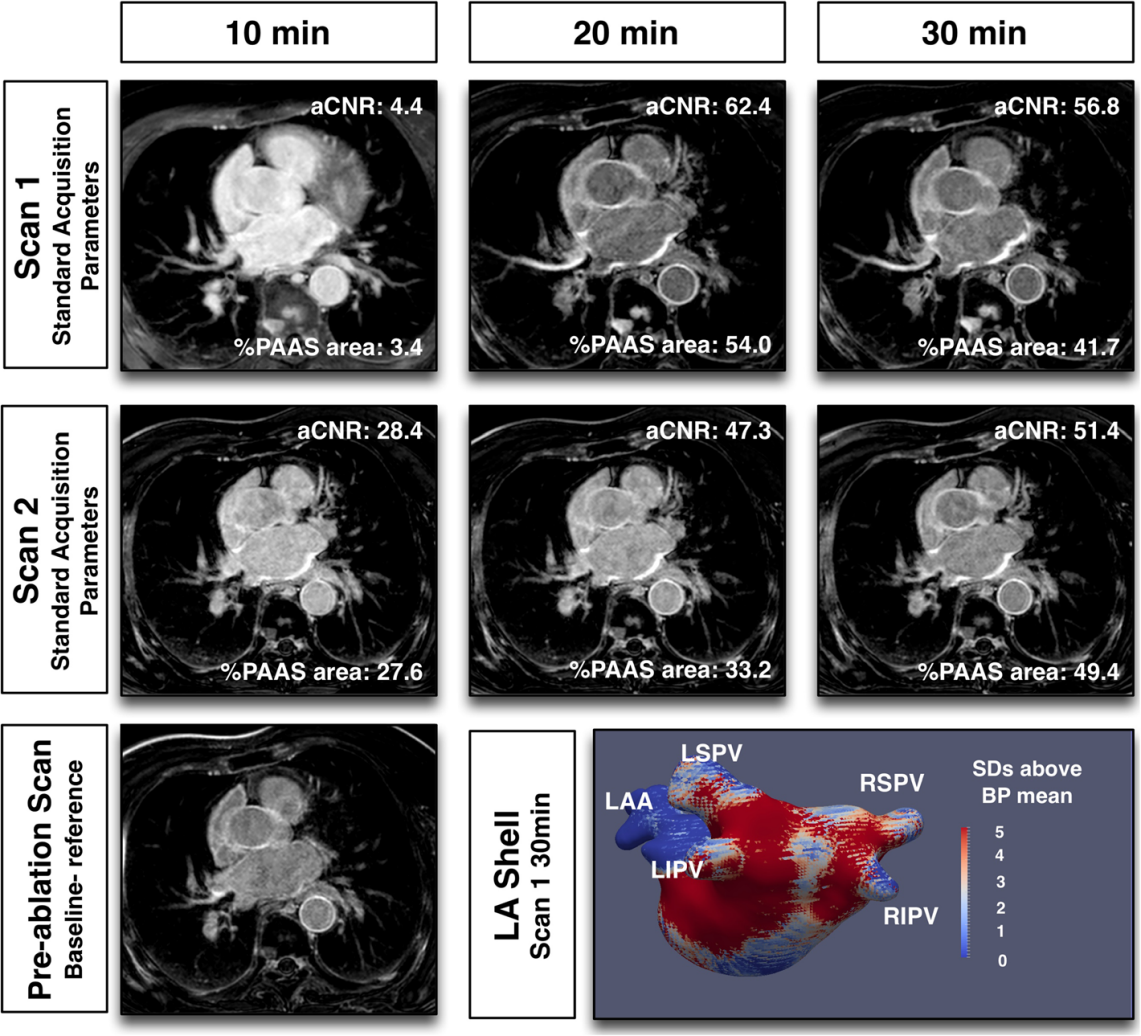
Typical imaging results with identical CMR scan protocol for scan 1 and 2. Note clearly enhanced regions in imaging performed at 30 min post gadolinium-based contrast agent administration, representing post-ablation atrial scar. Slight variations between scan sessions were common at 10 min, where contrast was changing most rapidly, but stabilised by 30 min

**Fig. 3:**
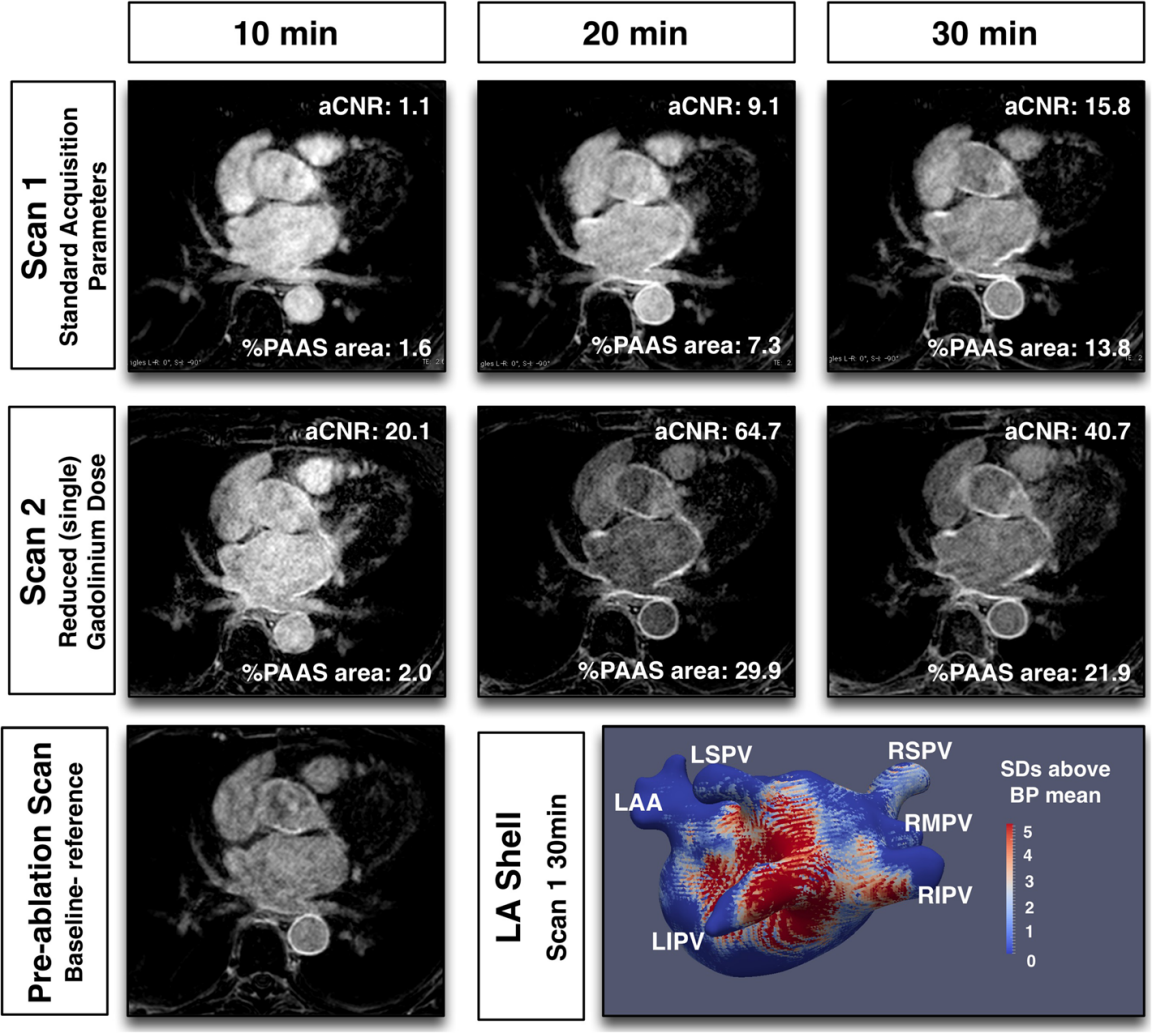
Typical imaging with reduced, single, gadolinium dose for Scan 2

**Fig. 4:**
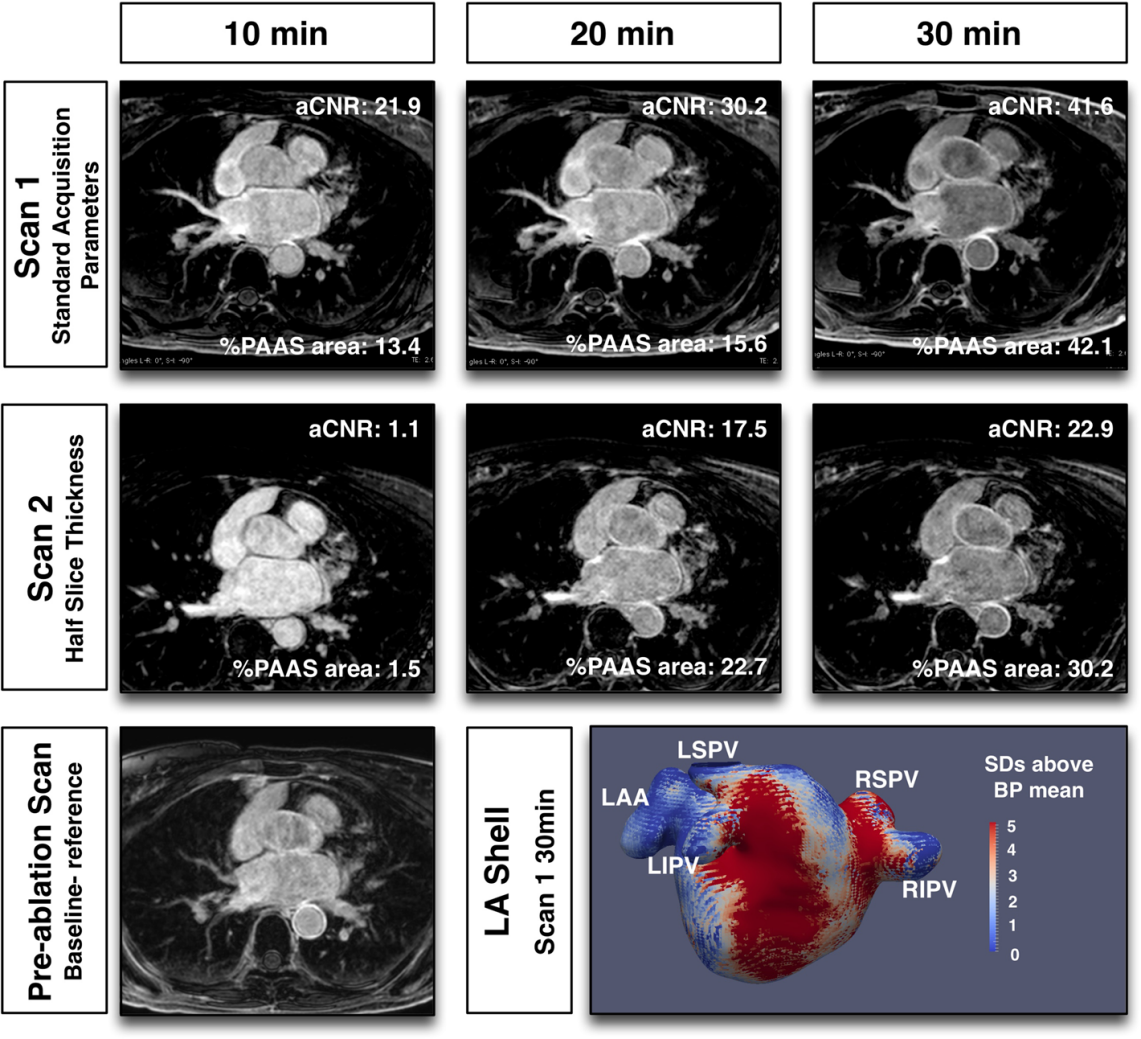
Typical imaging with half slice thickness for Scan 2

**Fig. 5:**
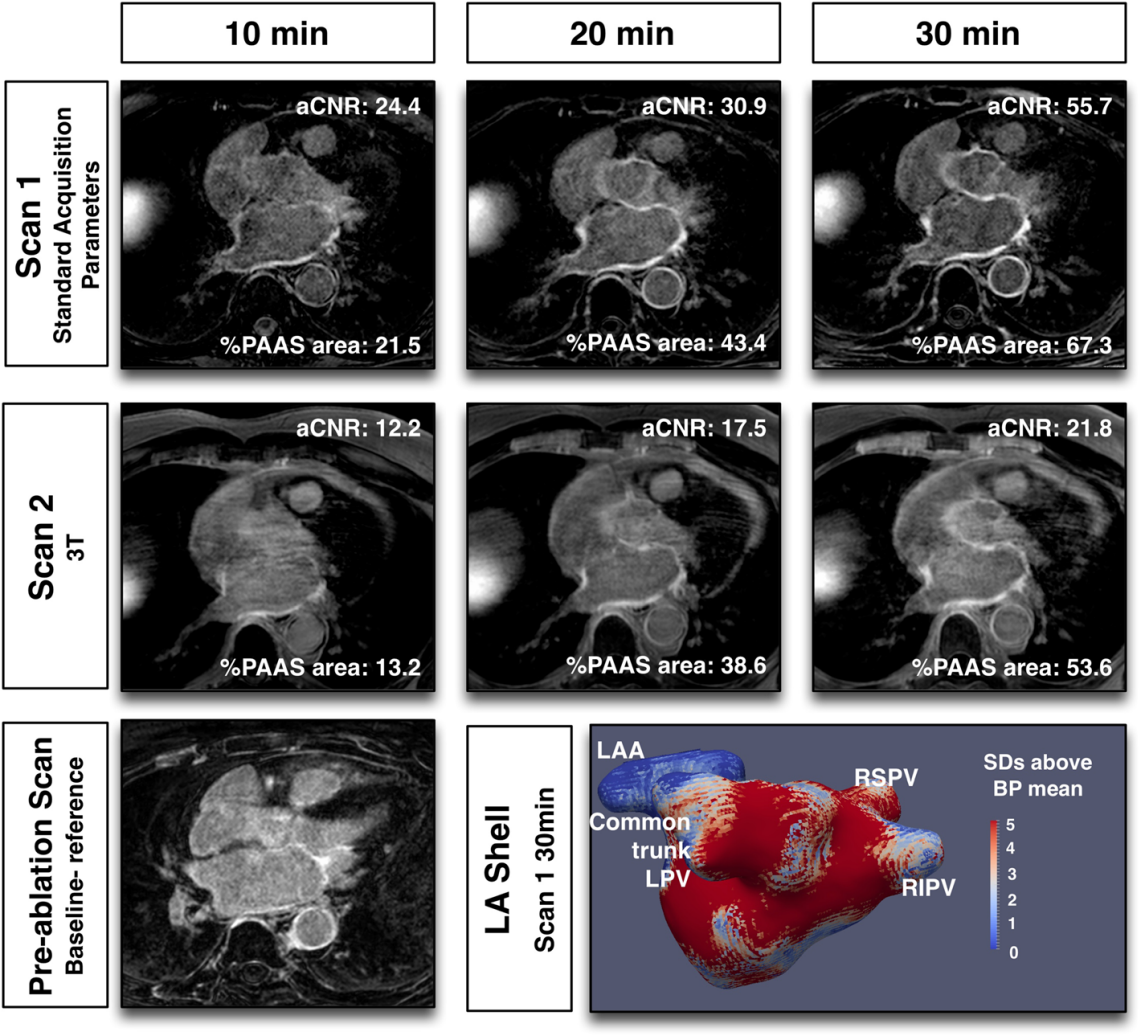
Typical CMR imaging with Scan 2 performed at 3 T

The CMR imaging sets for 40 subjects were evaluated. In total, there were 40 datasets (one per subject) acquired prior to ablation and 231 datasets post-acquisition (271 out of maximum possible 280, 96.7%). Typical imaging with each set of imaging parameters is illustrated in Figs. 2, 3, 4 and 5. Of the nine 3D LGE acquisitions not performed, three were at Scan 1 (patient tolerance), three at the standard scan 2 (single patient with viral illness), one at single gadolinium dose scan 2 (patient tolerance) and two from 3 T scan 2 (patient tolerance) (Fig. 1). Myocardial T1 time at scan 1 was 989 ± 21 msec, and at scan 2 was 987 ± 22 msec (paired t-test *p* = 0.34, subjects allocated to 3 T excluded).

Subject baseline demographics are summarised in Table 2. There were no significant differences between patients allocated to 1.5 T versus 3 T scanner.

**Table 2:** Baseline demographics, as assessed at the initial scan prior to ablation procedure

	All Subjects (*n* = 40)	Scan 2 1.5 T (*n* = 30)	Scan 2 3 T (*n* = 10)	*p*-value
Male	31 (78%)	22 (73%)	9 (90%)	0.27
Paroxysmal AF	20 (50%)	17 (56%)	3 (30%)	0.14
CHA_2_DS_2_VASC Score	1 (IQR 0–2)	1 (IQR 0–2)	0 (IQR 0–1.5)	0.28
AF duration (years)	3.0 (IQR 2.1–5.3)	2.5 (IQR 1.9–5.0)	5.5 (IQR 2.6–12.5)	0.19
Significant Comorbidities	22 (56%)	16 (53%)	6 (60%)	0.71
Age (years)	61 ± 10	61 ± 8	61 ± 13	0.99
Weight (kg)	88 ± 17	88 ± 18	87 ± 12	0.77
Height (cm)	176 ± 7.1	176 ± 6.4	177 ± 9.3	0.60
BMI (kg/m^2^)	28.4 ± 5.3	28.7 ± 5.9	27.6 ± 3.1	0.48
HR at baseline scan (bpm)	61 ± 10	61 ± 8	61 ± 13	0.99
Sinus rhythm at baseline scan	25 (62.5%)	19 (63%)	7 (70%)	0.70
LV ejection fraction (%)	60 ± 10	62 ± 10	58 ± 11	0.41
LA size (ml)	121 ± 32	122 ± 37	119 ± 19	0.75
LA fibrosis at baseline (%)	36.0 ± 13.9	36.7 ± 15.1	33.9 ± 9.3	0.49
LA ejection fraction (%)	30 ± 18	29 ± 19	34 ± 12	0.41
LV native T_1_ time at baseline scan (msec)	988 ± 22	991 ± 24	985 ± 21	0.33

*P*-value is for comparison between patients that underwent scan 2 in 1.5 T versus 3 T scanners

*LA* left atrium, *LV* left ventricle, *BMI* body mass index, *HR* heart rate, *bpm* beats per minute.LA fibrosis was determined on manual segmentation of the left atrial wall, and thresholded at an image intensity ratio of 0.97 to the blood pool mean- see Online Supplement for details [26]

### Likert scale assessment of imaging quality

**Fig. 6:**
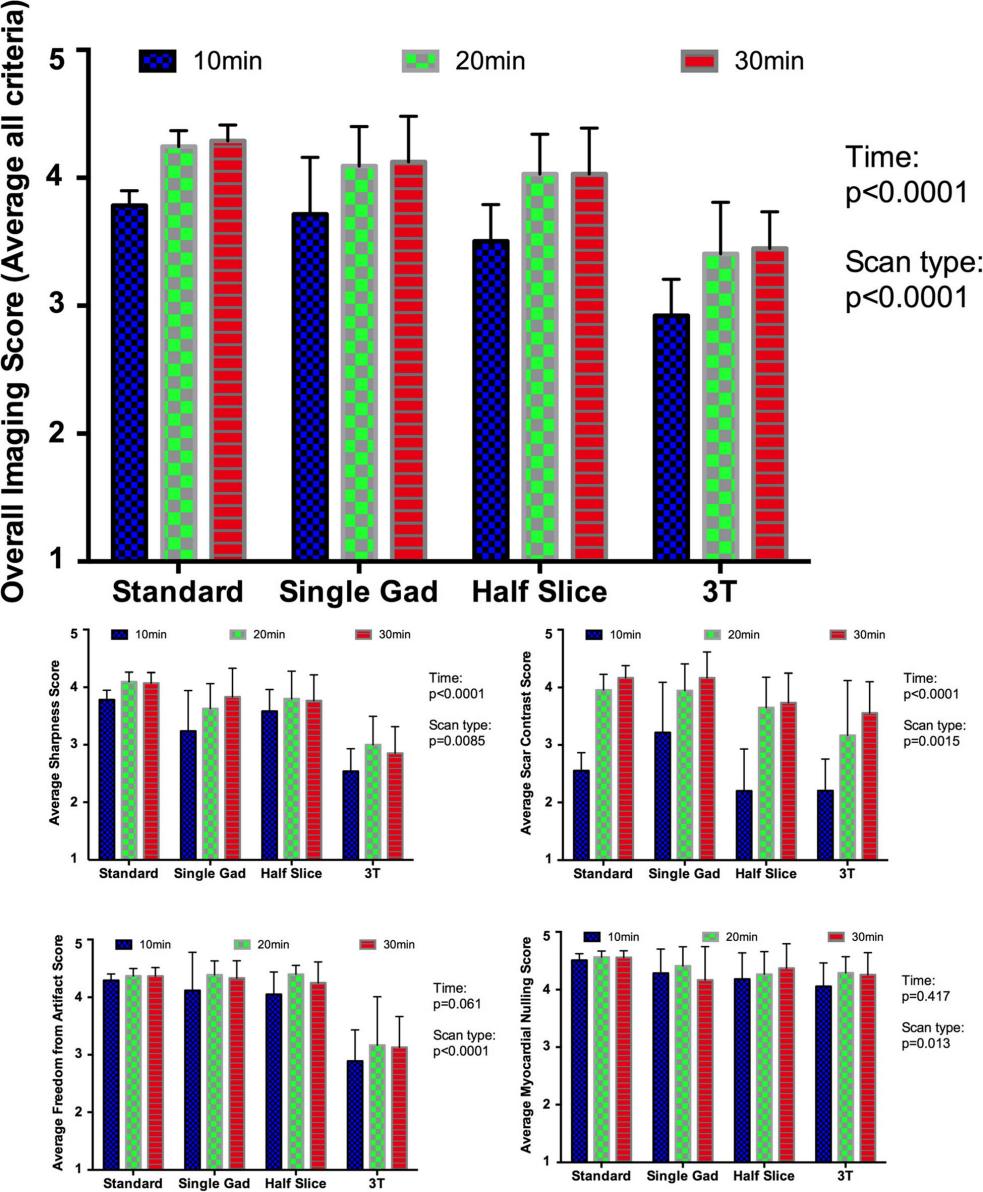
Summary of Likert Scores for each set of imaging acquisition parameters See above for full details of imaging parameters. The blue, green and red columns indicate imaging performed at 10 min, 20 min and 30 min post GBCA injection respectively. *P*-values for two-way ANOVA

Imaging quality in terms of sharpness, scar contrast and overall score improved with time from GBCA administration across all imaging parameter sets (*p* < 0.001), whilst freedom from artefact and quality of myocardial nulling remained unchanged (Fig. 6). Between imaging parameter sets, imaging at 3 T had a lower overall imaging score, primarily driven by inferior sharpness and increased artefact. Imaging quality scores for the other imaging parameter sets demonstrated no significant differences when corrected for multiple comparisons. A subanalysis of the comparisons between acquisitions at 20 min and 30 min only was also performed and found no significant difference in any comparison except for ‘Contrast’ when standard acquisition parameters (*p* = 0.012) or all acquisitions (*p* = 0.003) were compared (see Additional file 1: Table S1). Likert score assessment was evaluated for reliability, and there was generally good interobserver consistency (ICC = 0.888 (95% CI 0.862–0.910)) and excellent intraobserver agreement (ICC = 0.964 (95% CI (0.939–0.979)) (Table 3). Imaging acquisitions where myocardial nulling did not receive at least a score of 3 (‘Good’) from all three observers were excluded from further analysis (*n* = 7 out of 231, 3%).

**Table 3:** Inter- and intraobserver intraclass correlation coefficients. Brackets indicate 95% confidence intervals

Intraclass Correlation Coefficient	Sharpness	Scar Contrast	Freedom from Artefact	Myocardial Nulling	Total
Interobserver	0.763 (0.707–0.809)	0.892 (0.866–0.913)	0.789 (0.739–0.830)	0.824 (0.783–0.859)	0.888 (0.862–0.910)
Intraobserver	0.924 (0.872–0.954)	0.965 (0.942–0.979)	0.853 (0.755–0.912)	0.876 (0.786–0.927)	0.964 (0.939–0.979)

### Timing of LGE acquisition

Following GBCA administration, 3D LGE acquisitions were targeted to be commenced at 10, 20 and 30 min (performed at median 10 min (IQR 9-12 min), 22 min (IQR 20-24 min) and 32 min (IQR 30-35 min) respectively). The small drift in true rather than intended acquisition time reflects acquisition times > 10 min, and also operator time taken to compensate for inadequate respiratory navigator signal, respiratory gating efficiency, low heart rate, and patient-related delays such as anxiety. Total acquisition time for the sequence was broadly similar at all time intervals (6.4 ± 3.1 min at 10 min, 6.7 ± 2.5 min at 20 min and 7.3 ± 3.5 min at 30 min (*p* = 0.06, repeated measures ANOVA)) and there was no significant change in the estimator of noise (SD of lung: 3.3 ± 1.7 at 10 min, 3.2 ± 1.9 at 20 min, 3.0 ± 1.8 at 30 min (*p* = 0.31, repeated measures ANOVA)).

**Fig. 7:**
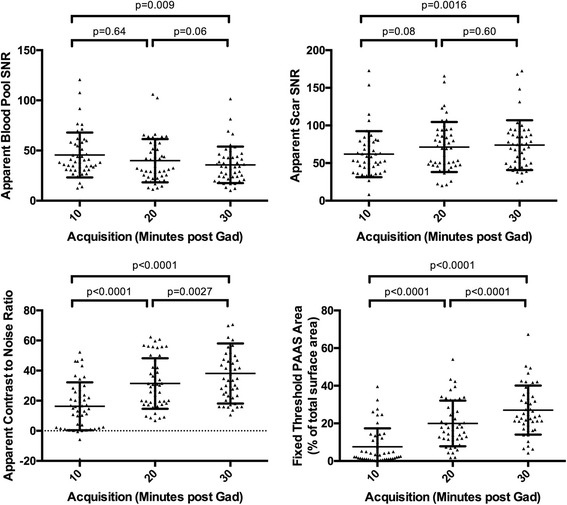
Relationship of acquisition timing post GBCA administration and signal/contrast-to-noise ratios. (Top left) apparent blood pool signal-to-noise ratio (SNR), (top right) apparent scar SNR, (bottom left) apparent scar to blood pool contrast-to-noise ratio, and (bottom right) LA scar area as a percentage of left atrium, thresholded at 3.3 standard deviations above blood pool mean. Values presented are for standard acquisition parameters only (*n* = 49 at each time point). For each plot, 3 *p*-values are presented: the top is p-value for Friedman test, assessing overall impact of time, and the bottom two are the p-values for Wilcoxon matched-pairs signed rank test

Figure 7 shows the effect of acquisition timing on blood pool aSNR, scar aSNR, aCNR and fixed threshold (3.3SD above blood pool mean) overall scar area (%PAAS), for standard acquisitions only (*n* = 49 at each time point, total 147 3D datasets). For all parameters there was a significant change with time: blood pool aSNR fell (*p* = 0.009), as scar aSNR, aCNR and scar area all increased (*p* = 0.0016, *p* < 0.001 and p < 0.001 respectively). For blood pool and scar aSNR, there was no significant change with Bonferroni correction between 20 and 30 min (median 32.6 (IQR 23.6–56.6) versus median 33.9 (IQR 23.8–43.3) (p = 0.06) and median 69.6 (IQR 47.2–93.9) versus median 71.4 (IQR 47.3–90.3) (*p* = 0.60) respectively). However, post-processing of atrial scar is heavily reliant on the interaction of these two factors, and both aCNR (median 29.3 (IQR 18.3–47.3) versus median 34.7 (IQR 23.2–48.5) (*p* = 0.0027)) and %PAAS area at fixed threshold (median 17.9% (IQR 11.7–28.5%) versus median 24.1% (IQR 19.2–35.2%) (*p* < 0.001)) were lower at 20 than 30 min.

**Fig. 8:**
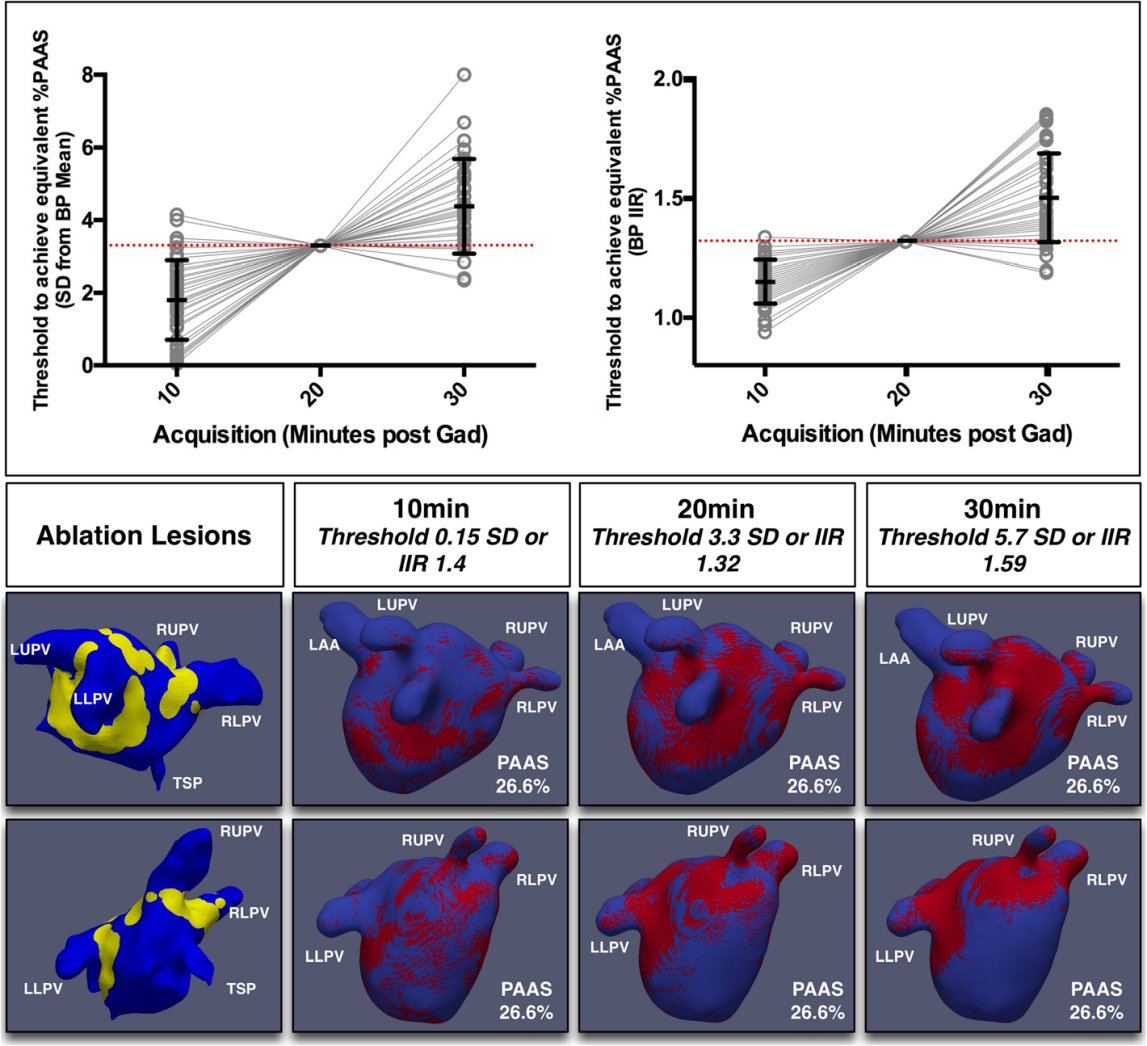
Impact of acquisition timing upon PAAS thresholding and quality of scar imaging. Charts (upper panels) show the change in threshold required for the acquisitions at 10 min and 30 min in order to achieve the same total %PAAS as that observed at 20 min at 3.3standard deviations (SD) above the blood pool (BP) mean (left- red line at 3.3) and BP image intensity ratio (IIR) 1.32 (right- red line at 1.32). Lower panels show the impact of changing time and threshold on scar location for a typical subject, with identical scar burden for all acquisitions. The true ablation lesion locations are shown in yellow on left, with thresholded scar in three subsequent panels to the right. Note clearest delineation of scar at 30 min. LUPV: left upper pulmonary vein (PV), RUPV: right upper PV, LLPV: left lower PV, RLPV: right lower PV, TSP: trans-septal puncture site

PAAS thresholds at 10 and 30 min post-GBCA were derived that achieved the same %PAAS as ‘standard’ thresholds at 20 min: these are shown in Fig. 8, again for standard parameters only. A markedly less stringent threshold was required at 10 min in order to achieve the same %PAAS as at standard threshold at 20 min (1.80 ± 1.09 SD from blood pool mean or IIR 1.15 ± 0.09 versus standard 3.3SD from blood pool mean or IIR 1.32 respectively (both *p* < 0.001)). Conversely, a more stringent threshold could be applied at 30 min whilst achieving the same scar burden (4.37 ± 1.30SD from blood pool mean or IIR 1.50 ± 0.19 (both *p* < 0.001)). The DSC at 20 min, assessing colocation of ablation lesions and scar, was 0.37 ± 0.11. This was significantly higher than the DSC observed at 10 min using threshold to achieve the same %PAAS (0.27 ± 0.12, *p* < 0.001), but lower than that observed at 30 min (0.39 ± 0.11, *p* = 0.02).

### Scan parameters

**Fig. 9:**
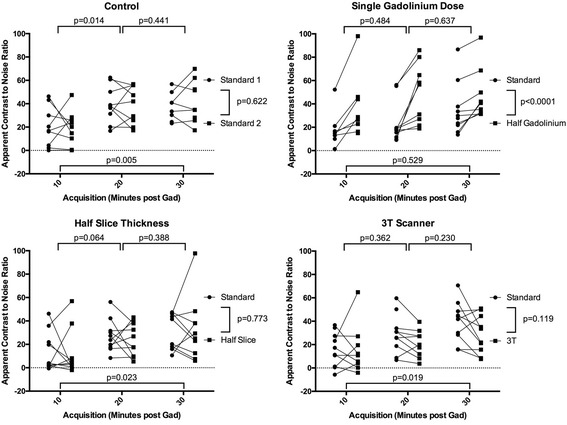
Impact of scan parameters on apparent scar to blood pool contrast-to-noise ratio. Paired acquisitions at 10, 20 and 30 min post GBCA injection, for control subjects (top left), reduced, single, GBCA dose (top right), half slice thickness (bottom left) and 3 T scanner (bottom right). Scan 1 (standard acquisition, circle) and scan 2 (experimental acquisition, square) are linked for each subject. P-values are for two-way repeated measures ANOVA: at the bottom of each plot is the p-value for variance with time, and to the right is the p-value for variance with acquisition parameter. Unpaired acquisitions are shown as unlinked circle or square and were not included in statistical analyses

The effect of scan parameters on aCNR are shown in Fig. 9. The effect upon blood pool and scar aSNR are shown in the online supplement (Additional file 2: Figure S1 and Additional file 3: Figure S2). Timing of the acquisition post GBCA administration remained an important determinant of aCNR across all imaging parameters (*p* < 0.005 to *p* = 0.023), except for the cohort randomised to single GBCA dose, where the impact of scan parameter dominated (time: *p* = 0.529, scan parameters: *p* < 0.001). There was no statistically significant interaction between scan parameters and acquisition timing for all analyses (*p* = 0.08 to *p* = 0.96).

For the control group, with identical scanning parameters in scan sessions 1 and 2, there was no significant difference between scan sessions. Single GBCA dose increased the aCNR (*p* < 0.001). At half slice thickness, there was no significant change in aCNR (*p* = 0.77). One potential factor to account for the absence of decrease in aCNR could have been the timing of the acquisitions, if half-slice acquisitions were significantly later due to over-run of the longer acquisition. However, there was no significant difference at any time point for scan 1 versus scan 2 acquisition commencement times (10 min: 11.6 ± 4 min versus 10.3 ± 1.4 min (*p* = 0.31), 20 min: 23.2 ± 5.4 min versus 23.3 ± 3.3 min (*p* = 0.95), 30 min 35.5 ± 6.8 min versus 34.1 ± 4.6 min (*p* = 0.51) respectively). For 3 T bore strength there was no significant difference in aCNR (*p* = 0.12).

A subanalysis of the impact of timing of acquisition was also performed with the 10 min acquisitions excluded, in view of the relatively poor imaging performance at this time point. aCNR did not differ significantly between 20 min and 30 min for any group.

## Discussion

The quality of 3D LGE imaging of PAAS varies widely between different scanning parameters. This has important implications for the routine performance of these scans and, in particular, clinical decision making on the basis of CMR-defined metrics. The findings are summarised as follows:

1. Imaging quality improves with time from GBCA administration and LGE imaging with standard parameters is best performed 30 min after injection of contrast
2. A reduced, single dose of GBCA (0.1 mmol/kg) improved aCNR without significant detrimental effect upon imaging quality
3. Halving slice thickness reduced the amount of PAAS detected at standard threshold
4. Imaging at 3 T magnet strength did not improve aCNR in this patient cohort, and was associated with a reduction in imaging quality and amount of scar detected

### Timing of acquisition

The finding that aCNR and scar detection increased with time is not surprising. In an informative study by Goldfarb et al. [28], the T_1_ values for LV myocardial scar, viable myocardium and blood pool at 2 min intervals following GBCA administration up to 1 h. Theyfound that the discrimination between scar and viable myocardium was significant even at very early acquisitions (< 10 min). However, the discrimination between blood pool and scar was only significant at > 10 min, and continued to improve with time, such that imaging at > 30 min was recommended for blood pool to scar differentiation.

For PAAS imaging, it is the blood pool to scar differentiation that is crucial, not viable myocardium to scar. All centers currently acquire 3D LGE imaging with in-plane resolution around 1.3 × 1.3 mm (Table 1), and therefore blood pool partial voluming effects are inevitable for most voxels within an atrial wall of thickness 2-4 mm [29, 30]. PAAS detection will be improved as the blood pool signal falls, regardless of the image interrogation technique. This is most critical when the maximum intensity projection technique is used to interrogate scar [3, 8–10], but the principle also applies for a voxel-by-voxel interrogation of the atrial wall [2, 7, 11, 31].

In addition, it should be noted that the ‘true’ threshold for atrial scar, if it is even appropriate to binarise scar and healthy atrial myocardium at all, almost certainly changes with timing of acquisition. 3.3 SD from the blood pool mean was selected as an objective threshold with histological validation [13], but those pre-clinical scans were only performed at approximately 20 min post GBCA administration, and the confidence intervals were wide. Likewise, an equivalent threshold of IIR 1.32 at 20 min was also selected in view of demonstrated clinical correlation, but the derivation of the threshold was from ventricular myocardial imaging [24].

Clearly, there will be a tight correlation between aCNR and %PAAS at a fixed threshold, and the increase in %PAAS scar detected with time should be interpreted with caution. At earlier acquisitions, the ‘true’ threshold, representing an optimal compromise of sensitivity and specificity, is likely to be lower as the scar signal intensity lies closer to that of the blood pool. Instead, increasingly high thresholds may be best viewed as an index of the confidence with which portions of the atrial shell may be classified as neither blood pool nor healthy myocardium. The innate advantage of the maximum intensity projection technique and the ‘z-score’-type threshold is that it provides a degree of quantification of the likelihood that a signal intensity on the shell could possibly be derived from a blood pool voxel. Further work is required in order to define the appropriate thresholds for delineation of PAAS at different timings post GBCA, but this study has suggested that there is a decrease in the level of noise in scar detection with time from GBCA administration. The lower panels of Fig. 8 show that areas that have not been ablated may be inappropriately delineated as scar at early acquisitions. Later acquisitions, using a higher threshold, are less likely to detect coincidental noise thus increasing confidence in scar (higher specificity for PAAS detection at later acquisitions). The improvement in DSC, reflecting appropriate scar detection, from 20 min to 30 min was small but significant.

The DSC values obtained in this study are lower than those found in a benchmark study comparing post-ablation scar locations to expert-derived pseudo-truth (0.72–0.85 for algorithm-derived scar, 0.14–0.59 for fixed thresholds) [32]. However, it is important to consider that there are two additional factors that will contribute to a DSC lower than those achieved for analysis of PAAS on the same raw image. Firstly, for this study PAAS was compared to ablation on a different LA anatomical shape, acquired on EAM. There will inevitably be regions of mis-registration of the relatively narrow bands of scar. Secondly, scar location is clearly not perfectly recorded on EAM. An average location of energy delivery is recorded, but is confounded by contact force, stability, and other determinants of scar formation. Therefore, many locations where energy is delivered will not have true scar formation. An average DSC of 0.39 is therefore in the anticipated range for a good assessment of ablation scar.

The timing of 3D LGE acquisition varies widely in published studies, with no center routinely imaging at > 30 min (Table 1), although it should be noted that some vendors such as Siemens Healthineers routinely acquire central k-space in the middle, rather than start, of the scan, effectively pushing the scan acquisition timing back by half of the scan duration compared to this study. There have been two large non-selective studies of PAAS imaging, where CMR imaging was performed regardless of recurrence status. Both acquired 3D LGE sequences at around 15 min post-GBCA administration and gaps in ablation lines were frequently detected. Badger and co-workers detected gaps in PV scar at 405/576 veins (70%) and in 93% of patients overall [3]. Akoum and co-workers, on assessment of a subset of the DECAAF study, detected circumferential scar at 1.26 veins per patient (gaps estimated at 67% of veins) and also in 93% of patients overall [2]. The findings of the present study suggest that the incidence of gaps may hypothetically have been substantially lower if image acquisition had been delayed until later after GBCA administration.

### Scan parameters

The improvement in PAAS imaging with reduced, single, GBCA dose relates largely to the increase in blood-pool-to-scar-contrast, and the superiority is most marked at early acquisitions. The persistence of the improved aCNR at 20 and 30 min is an interesting illustration of the aphorism that ‘less is more’ and is not necessarily apparent from first principles: this is an important finding of this study. The relationship between contrast concentration and signal intensity is not a linear one, and the halving of contrast concentration in any given compartment does not necessarily result in a halving of relaxation rate (the inverse of the relaxation time constant). Similarly, the increase in signal resulting from a shortened T_1_ time-constant is a relationship that is also highly dependent upon inversion time and repetition time [33]. Furthermore, the time dependent concentrations of GBCA within the blood pool and atrial myocardial scar compartments have not been clearly ascertained [33].

The lack of improvement in imaging at 3 T may be explained at least in part by challenges in image acquisition that are more frequently encountered in this environment. ECG interference is higher, leading to triggering errors, and the respiratory navigator is less reliable, although once successfully commenced overall acquisition time was unchanged from controls. Contrast behavior is also relatively unchanged, with minimal reduction in relaxivity of GBCAs at higher field strengths (5.0 mmol^− 1^ s^− 1^, (range 4.7–5.3 mmol^− 1^ s^− 1^) at 3 T in plasma, versus 5.2 mmol^− 1^ s^− 1^ (range 4.9–5.5 mmol^− 1^ s^− 1^) at 1.5 T) [34]. However, the acquisition window was late atrial diastole (onset 296 ± 40 msec post R-wave), which was more frequently impinged upon at the longer inversion times necessary for imaging at 3 T, requiring compromise in terms of acquisition window. The mean inversion times at 10 min, 20 min and 30 min were 238 msec, 267 msec and 288 msec respectively, and the acquisition window had to be delayed for one subject at 10 min, two at 20 min and four at 30 min. Generally the impingement upon the window was only by 10-20 msec, but the maximum impingement for any subject was 80 msec, markedly increasing the nominal acquisition time. Finally, many of the acquisition parameters clearly cannot be directly transposed from the 1.5 T to 3 T environment, and in particular compromises regarding receiver bandwidth, TE and TR had to be made, which may also have impacted upon imaging quality.

There was a general decline in imaging quality with half-slice thickness, which is not surprising. The reduced voxel size will decrease the voxel SNR, but on direct image assessment the blood pool and scar aSNR remained relatively preserved, as was aCNR (Additional file 2: Figure S1 and Additional file 3: Figure S2). However, when defining scar tissue at 3.3SD above the blood pool mean there was a significant decrease in PAAS area overall. This has implications for the detection of small gaps. In the recent study by Bisbal et al., they found median gap size of 13 mm, but the smallest was 1.6 mm [7], and Ranjan et al. detected deliberate gaps as small as 1.4 mm, using a 1.0 × 1.0 × 1.5 mm resolution 3D LGE acquisition in an animal model [14]. Small gaps will only be detectable within plane for thicker slice 3D acquisitions, and not if the gap lies between slices. Two consecutive orthogonal acquisitions may represent the best compromise for accurate gap detection whilst maintaining scar sensitivity, but would require more complex registration and co-processing for gap detection.

### Clinical implications

PAAS imaging in the immediate term presents opportunities for non-invasive evaluation of conventional and novel therapies. This includes assessment of the impact of contact force [35], evaluation of ablation extent by cryoballoon [36], and even ablation-induced modification of fat pads containing ganglionated plexi [37]. Optimal, and ideally uniform, imaging acquisition parameters would increase precision and facilitate comparison of studies.

The use of PAAS imaging to guide ablation procedures is more controversial. Interscan reproducibility needs to be demonstrated, and sensitivity needs to improve. However, if the findings of Bisbal et al. can be replicated then there is opportunity for swifter and more efficacious re-do procedures [7]. This may become even more relevant in the light of the PRESSURE trial where it was demonstrated that there was increased arrhythmia free survival with prophylactic repeat ablation procedure at 2 months, regardless of recurrence status [38]. Non-invasive CMR correlates that identify subjects who would benefit from pre-emptive repeat procedures could be extremely valuable.

For post-ablation macro re-entry arrhythmias, identification of PAAS may also assist in the pre-procedural prediction of the arrhythmia mechanism. This in turn may inform activation mapping strategy, diagnostic manoeuvres and possibly lesion delivery. Zahid et al. used LA LGE datasets to derive patient specific models of LA tachycardia pathways, in combination with fibre orientation atlas. In 7 out of 10 patients (all post–PVI) it was possible to model a LA macro-reentrant circuit, and the ablation trajectory that was successful clinically was predicted in-silico in all 7 patients [39].

### Limitations

This study was performed at 3 months post ablation, using Gadovist as the GBCA, and is an evaluation of chronic scar formation. As such, the results are not directly applicable to the assessment of acute lesion formation, and could not be used to guide acute repeat ablation during the index procedure in a hybrid-type environment. Likewise, there is evidence that there is a slow fading of scar with time [40], and the application of these results to imaging > 3 months post-ablation, or using different contrast agents, should be performed with caution.

There is no gold standard for validation of PAAS detection, in the absence of histological assessment. Manual segmentation was considered, but strongly relies upon subjective user-defined thresholding of the scar and was therefore rejected. Voltage mapping has been only weakly correlated with PAAS, and it is likely that registration errors, bipolar sampling considerations and electrode size confer upon voltage mapping a similar level of error as CMR assessment of scar. Furthermore, there is evidence that voltage and true scar are only moderately well-correlated [19]. Therefore, the study has focused on optimising sensitivity, rather than evaluations of specificity of scar detection.

In terms of the study design and the analysis of the impact of the timing of the acquisition post-gadolinium, it is important to note that the study is underpowered to detect small differences with time from GBCA administration for the non-standard acquisition parameters. In addition, the acquisitions could not be performed at identical timepoints post-GBCA administration, which may introduce a bias for late acquisition for patients that experienced more difficult and prolonged imaging acquisitions. On account of technical considerations, it was not possible to randomize patients to the 3 T scanner, and additionally scanning parameters cannot be replicated exactly between different scanner field strengths. The 3 T acquisition parameters aimed for equivalence, rather than optimisation for 3 T imaging alone, and the results at the higher field strength should be interpreted with caution. The interval between scan sessions was minimized in order to control for time dependent scar maturation [40], but there was a possibility of residual GBCA accumulation between scans. T_1_ relaxation times for the myocardium were unchanged between scan sessions, and there was no systematic difference between scans in any parameter for control patients. Recent studies have suggested that very low concentrations may persist beyond 48 h [41], despite the interval being > 20 half-lives, but the impact on the results is likely to be minimal.

## Conclusions

Qualitative image quality (sharpness, scar contrast and overall quality score) improves with time after GBCA administration (from 10 min to 30 min). Quantitatively, blood pool aSNR decreases while scar aSNR, aCNR and fixed-threshold %PAAS increase. In a sub-analysis comparing acquisitions at 30 min and 20 min post GBCA, both qualitative and quantitative measures of CNR were higher at 30 min. A reduced, single, dose of GBCA is superior in terms of contrast-to-noise ratio, whilst reduced slice thickness and imaging at 3 T tend to result in inferior imaging quality.

## Additional files

Additional file 1: Details of acquisition and analysis techniques. (DOCX 26 kb)

Additional file 2: **Figure S1.** Impact of scan parameters on blood pool apparent signal to noise ratio. Paired acquisitions at 10, 20 and 30 min post GBCA injection, for control subjects (top left), half GBCA dose (top right), half slice thickness (bottom left) and 3 T scanner (bottom right). Scan 1 (standard acquisition, circle) and scan 2 (experimental acquisition, square) are linked for each subject. *P*-values are for two-way repeated measures ANOVA: at the top of each plot is the *p*-value for variance with time, and to the right is the p-value for variance with acquisition parameter. Unpaired acquisitions are shown as unlinked circle or square, and were not included in statistical analyses. (JPEG 268 kb)

Additional file 3: **Figure S2.** Impact of scan parameters on scar apparent signal to noise ratio. Paired acquisitions at 10, 20 and 30 min post GBCA injection, for control subjects (top left), half GBCA dose (top right), half slice thickness (bottom left) and 3 T scanner (bottom right). Scan 1 (standard acquisition, circle) and scan 2 (experimental acquisition, square) are linked for each subject. P-values are for two-way repeated measures ANOVA: at the top of each plot is the p-value for variance with time, and to the right is the p-value for variance with acquisition parameter. Unpaired acquisitions are shown as unlinked circle or square, and were not included in statistical analyses. (JPEG 286 kb)

## Abbreviations

aCNR: Apparent contrast-to-noise ratio
AF: Atrial fibrillation
aSNR: Apparent signal-to-noise ratio
bSSFP: Balanced steady state free precession
CI: Confidence interval
CMR: Cardiac magnetic resonance
CNR: Contrast-to-noise ratio
DSC: Dice similarity coefficient
EAM: Electroanatomic mapping
ECG: Electrocardiogram
GBCA: Gadolinium based contrast agent
ICC: Intraclass correlation coefficient
IQR: Interquartile range
IR: Inversion recovery
LA: Left atrium/left atrial
LGE: Late gadolinium enhancement
LV: Left ventricle/left ventricular
MIP: Maximum intensity projection
PAAS: Post-ablation atrial scar
PV: Pulmonary vein
RF: Radiofrequency
ROI: Region of interest
RV: Right ventricle/right ventricular
SD: Standard deviation
SI: Signal intensity
SNR: Signal-to-noise ratio
TE: Echo time
TR: Repetition time
WACA: Wide area circumferential ablation
