# Supplementary material for: Optimization of late gadolinium enhancement cardiovascular magnetic resonance imaging of post-ablation atrial scar: a cross-over study
Source: J Cardiovasc Magn Reson. 2018 May 3;20:30. doi: 10.1186/s12968-018-0449-8 (PMC5932811; doi:10.1186/s12968-018-0449-8)
Supplement: Supplementary file 1 — Details of acquisition and analysis techniques. (DOCX 26 kb) [file 12968_2018_449_MOESM1_ESM.docx]

Online Supplement

Fibrosis Assessment

Atrial fibrosis analysis was performed on an MITK-based platform (German Cancer Research Centre, Heidelberg, Germany), with custom-build modifications to enable the quantification of atrial fibrosis. The LA endocardial surface was defined via manual segmentation within the 3D LGE volume on a slice by slice basis, using 3D interpolation to minimise slice-by-slice discontinuities. A 2mm surface dilation was used to define the epicardial border, in keeping with established methods (1), and a mean intensity projection technique through the defined atrial wall was used to ascribe a single signal intensity value to each point on the LA endocardial surface model (typically 20,000 polygons per shell). The mitral valve, distal pulmonary veins (2mm distal to antrum) and LA appendage were removed using the Clip filter in Paraview (Kitware, New York, NY, USA) and the surface was re-extracted as a binary file.

LA scar burden was quantified using an image intensity ratio threshold (0.97 times mean blood pool (BP) signal intensity (1)). BP signal intensity was measured for a 4ml spherical volume placed in the center of the LA blood pool, distant from artefact including respiratory navigator signal.

Scar Quantification

For post-ablation scans, a gated magnetic resonance angiogram (GMRA) 3D dataset was acquired as a high contrast template, delineating the LA endocardial border. The acquisition was commenced 90seconds after the start of a slow infusion of GBCA at 0.3ml/second (2). (3D inversion recovery spoiled gradient echo acquisition with coverage identical to that of subsequent LGE acquisitions, to include the whole of the LA in axial orientation. TR 5.5msec, TE 3.0msec, flip angle 25°, low-high k-space ordering, increased receiver bandwidth (890Hz, fat-water ratio 0.5), respiratory and ECG gated (end atrial diastole, maximum 120msec window, identical to LGE acquisitions), 1.3x1.3x4mm^3^ with 2mm slice overlap (typically 50 slices per acquisition), SPIR fat suppression, empirical inversion time 200msec).

Scar quantification was then performed on the same MITK-based platform. For the LA segmentation, the GMRA was used to derive the atrial mask, and the segmentation was performed using the ‘Region Growing Tool’ with 3D interpolation on the MITK platform with ‘Segmentation Plugin’ (German Cancer Research Center, Division of Medical and Biological Informatics, Im Neuenheimer Feld 280, 69120 Heidelberg, Germany). Where a GMRA acquisition was inadequate (n=5), manual segmentation was performed on a slice-by-slice basis on the same platform.

The LA template was then applied to the LGE acquisitions, registering the GMRA to LGE acquisitions to ensure optimal alignment. The registration was performed as a rigid registration (3), with six degrees of freedom (3 translations and 3 rotations). The derived transformation of the GMRA image onto the LGE image was then applied to the segmentation, and quality of registration was confirmed visually.

Image interrogation was performed using a maximum intensity projection technique (3mm external and 1mm internal). The 3mm external interrogation was based upon the maximum typical atrial wall thickness (4), and the smaller internal projection was performed in order to avoid detection of respiratory navigator artefact whenever possible. Atrial shells were exported as a .vtk file, with a raw image intensity unit recorded at each surface triangle of the shell.

Supplementary Results

|  | Standard | Half Gad | Half Slice | 3T | All |
| --- | --- | --- | --- | --- | --- |
| **Total Likert** | 0.13 | 0.97 | 0.99 | 0.46 | 0.15 |
| **Sharpness** | 0.91 | 0.36 | 0.87 | 0.73 | 0.95 |
| **Contrast** | **0.012** | 0.69 | 0.51 | 0.17 | **0.003** |
| **Freedom from Artifact** | 0.87 | 0.25 | 0.34 | 0.99 | 0.39 |
| **Myocardial nulling** | 0.82 | 0.24 | 0.37 | 0.47 | 0.99 |

Table 1. P-values for sub-analysis of Likert scores between acquisitions performed at 20min and 30min only.Wilcoxon Signed Rank test

References

1. Khurram IM, Beinart R, Zipunnikov V, Dewire J, Yarmohammadi H, Sasaki T, Spragg DD, Marine JE, Berger RD, Halperin HR, Calkins H, Zimmerman SL, Nazarian S. Magnetic resonance image intensity ratio, a normalized measure to enable interpatient comparability of left atrial fibrosis. *Heart Rhythm*. Elsevier; 2014;**11**:85–92.

2. Groarke JD, Waller AH, Vita TS, Michaud GF, Carli MF Di, Blankstein R, Kwong RY, Steigner M. Feasibility study of electrocardiographic and respiratory gated, gadolinium enhanced magnetic resonance angiography of pulmonary veins and the impact of heart rate and rhythm on study quality. *J Cardiovasc Magn Reson*. 2014;**16**:43.

3. Denton ER, Sonoda LI, Rueckert D, Rankin SC, Hayes C, Leach MO, Hill DL, Hawkes DJ. Comparison and evaluation of rigid, affine, and nonrigid registration of breast MR images. *J Comput Assist Tomogr*. 1999;**23**:800–805.

4. Platonov PG, Ivanov V, Ho SY, Mitrofanova L. Left Atrial Posterior Wall Thickness in Patients with and without Atrial Fibrillation: Data from 298 Consecutive Autopsies. *J Cardiovasc Electrophysiol*. Blackwell Publishing Inc; 2008;**19**:689–692.

5. Harrison JL, Jensen HK, Peel SA, Chiribiri A, Grøndal AK, Bloch LØ, Pedersen SF, Bentzon JF, Kolbitsch C, Karim R, Williams SE, Linton NW, Rhode KS, Gill J, Cooklin M, Aldo Rinaldi C, Wright M, Kim WY, Schaeffter T, Razavi RS, O’Neill MD. Cardiac magnetic resonance and electroanatomical mapping of acute and chronic atrial ablation injury: a histological validation study. *Eur Heart J*. 2014;**35**:1486–1495.
