# Supplementary figures and images for: Optimization of late gadolinium enhancement cardiovascular magnetic resonance imaging of post-ablation atrial scar: a cross-over study
Source: J Cardiovasc Magn Reson. 2018 May 3;20:30. doi: 10.1186/s12968-018-0449-8 (PMC5932811; doi:10.1186/s12968-018-0449-8)

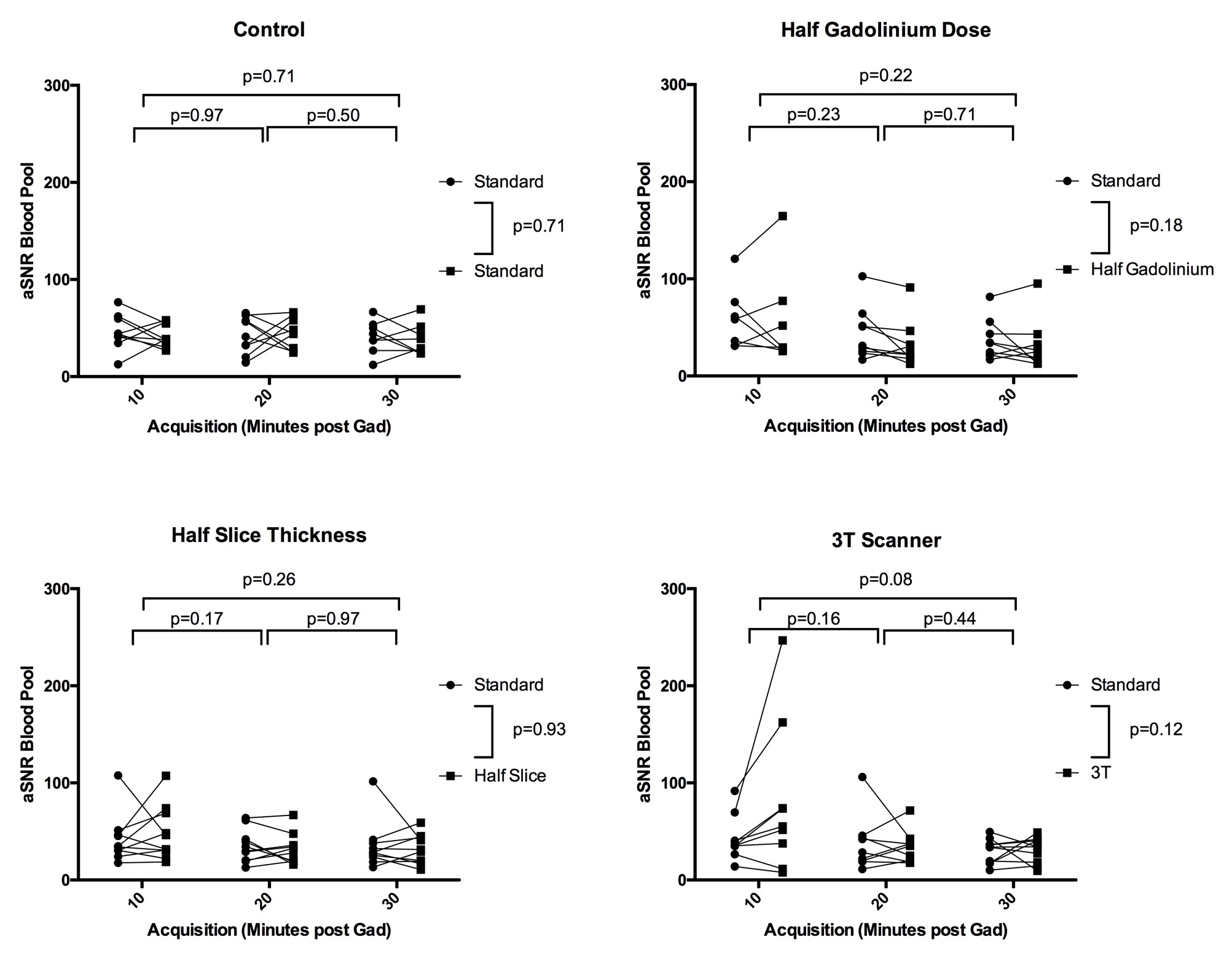

Supplement: Supplementary file 2 — Figure S1. Impact of scan parameters on blood pool apparent signal to noise ratio. Paired acquisitions at 10, 20 and 30 min post GBCA injection, for control subjects (top left), half GBCA dose (top right), half slice thickness (bottom left) and 3 T scanner (bottom right). Scan 1 (standard acquisition, circle) and scan 2 (experimental acquisition, square) are linked for each subject. P-values are for two-way repeated measures ANOVA: at the top of each plot is the p-value for variance with time, and to the right is the p-value for variance with acquisition parameter. Unpaired acquisitions are shown as unlinked circle or square, and were not included in statistical analyses. (JPEG 268 kb) [file 12968_2018_449_MOESM2_ESM.jpg]

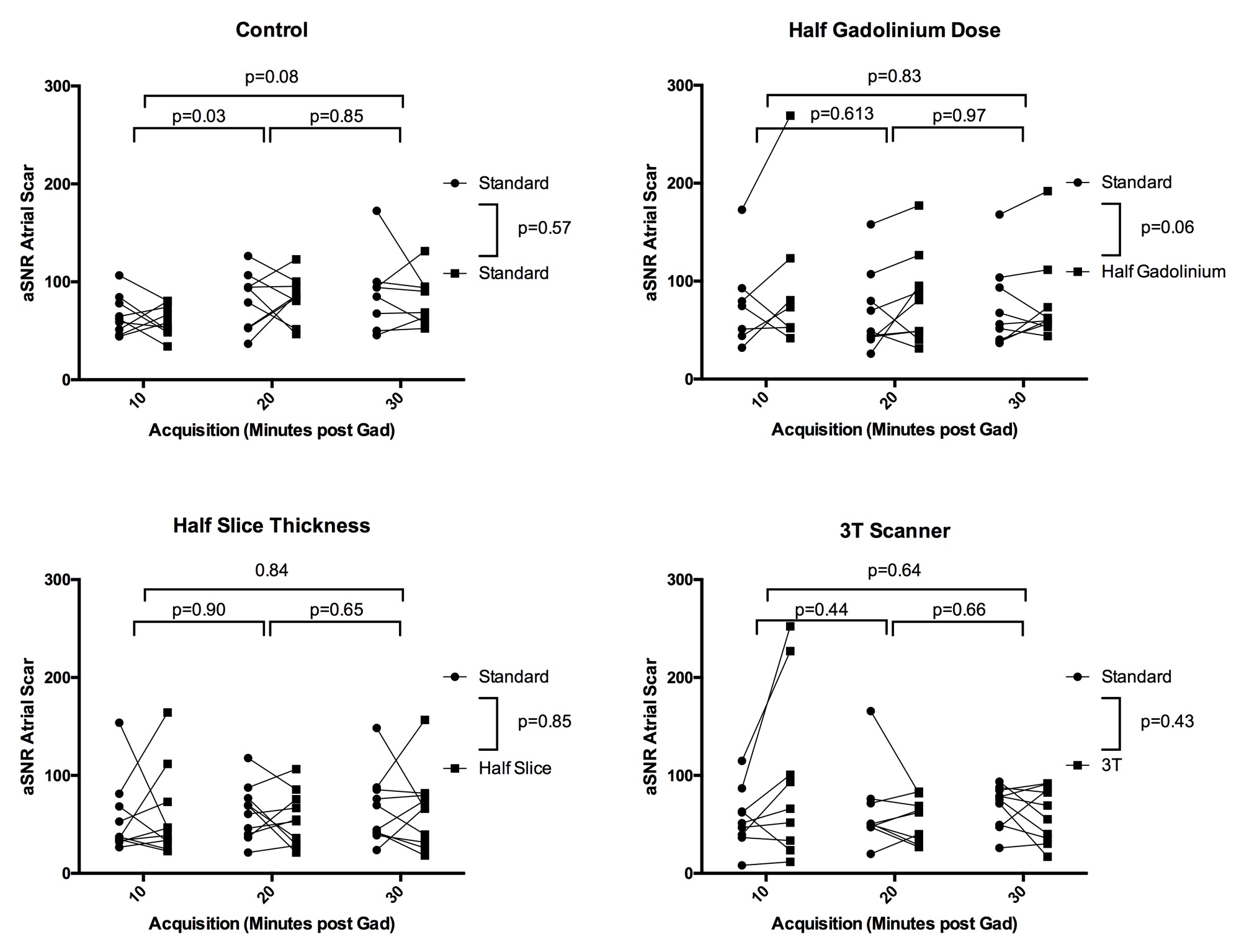

Supplement: Supplementary file 3 — Figure S2. Impact of scan parameters on scar apparent signal to noise ratio. Paired acquisitions at 10, 20 and 30 min post GBCA injection, for control subjects (top left), half GBCA dose (top right), half slice thickness (bottom left) and 3 T scanner (bottom right). Scan 1 (standard acquisition, circle) and scan 2 (experimental acquisition, square) are linked for each subject. P-values are for two-way repeated measures ANOVA: at the top of each plot is the p-value for variance with time, and to the right is the p-value for variance with acquisition parameter. Unpaired acquisitions are shown as unlinked circle or square, and were not included in statistical analyses. (JPEG 286 kb) [file 12968_2018_449_MOESM3_ESM.jpg]
